# Mindfulness Facets, Nature Relatedness, and Eco-Anxiety: Evidence from a Hungarian Adult Sample

**DOI:** 10.1192/j.eurpsy.2026.12219

**Published:** 2026-07-31

**Authors:** R. Szegedi, F. Mónus, Á. Bernáth, J. Kovács

**Affiliations:** Institute of Psychology, University of Debrecen, Debrecen, Hungary

## Abstract

**Introduction:**

Eco-anxiety—though not a formal diagnosis—captures climate-related distress overlapping with anxiety-spectrum processes. While mindfulness is often framed as protective, *facet-level links* to eco-anxiety are underexplored in Central/Eastern Europe. Ecological connectedness may also heighten sensitivity to environmental threat. We examined how mindfulness facets and nature relatedness jointly predict eco-anxiety.

**Objectives:**

(1) Test correlations between mindfulness and eco-anxiety; (2) identify protective vs risk-enhancing mindfulness facets; (3) probe indirect effects via well-being and nature relatedness.

**Methods:**

Hungarian adults (N=351; target N≈450; No of ethics approval UD-IP-2023/156) completed validated self-reports: FFMQ facets (Baer et al. *Assessment* 2006;13:27–45), EAQ-22 (Ágoston et al. *Climate Risk Management* 2022;37), PERMA positive composite (Butler & Kern *Int J Wellbeing* 2016;6:1–48), and NR-6 (Nisbet & Zelenski *Front Psychol* 2013;4). Linear models predicted eco-anxiety from five FFMQ facets (Nonjudging, Acting with Awareness, Nonreactivity, Describing, Observing), controlling for age and gender. Indirect effects via PERMA and NR were tested with 5,000-sample bootstraps. Reliability: FFMQ facets α=.49–.83 (Awareness lower; 3 items); EAQ-22 α=.93; NR-6 α=.88; PERMA positive (15 items) α=.89. Reporting & diagnostics: Standardized β with 95% CIs; collinearity acceptable (max VIF = 2.27).

**Results:**

Eco-anxiety correlated modestly and negatively with overall mindfulness (r = –.18). In the facet-only model (controls: age, gender), R²=.155: Awareness (β=−.23, 95% CI −.34 to −.11, p<.001) and Nonjudging (β=−.16, −.27 to −.06, p=.003) predicted lower eco-anxiety; Observing (β=+.12, .02 to .22, p=.024) predicted higher eco-anxiety; Describing/Nonreactivity were ns. Women reported higher eco-anxiety than men (β=.15, .04 to .25, p=.008). In the full model adding PERMA positive and NR (R²=.221), NR emerged as a robust positive predictor (β=.29, .18 to .40, p<.001), while Awareness (β=−.24, −.35 to −.13, p<.001) and Nonjudging (β=−.16, −.27 to −.06, p=.003) remained protective; Observing attenuated to ns and PERMA positive was ns. Indirect effects (pre-registered exploratory): Observing has a small–moderate, significant indirect via NR (ab_std≈.13, 95% CI .08–.20); other indirects trivial (|ab_std|≤.03; CIs include 0).

**Conclusions:**

This first facet-level test in a CEE sample suggests that apparent protective effects of some mindfulness facets attenuate once nature relatedness is considered. Ecological connectedness emerges as a double-edged factor—supporting environmental identification yet sensitizing to climate-related distress—highlighting both mindfulness dispositions and connectedness as clinically relevant but complex intervention targets. Preliminary data are presented; full-sample analyses (N≈450) will be reported at the conference.

**Disclosure of Interest:**

None Declared

